# Impact of nutritional status and abnormal bone–muscle metabolism on chronic low back pain after lumbar decompression surgery: a multicenter predictive model study based on paraspinal muscle parameters

**DOI:** 10.3389/fnut.2026.1848387

**Published:** 2026-07-02

**Authors:** Shihao Zhou, Zhenqian Qi, Xiaowan Xu, Hongshun Zhao, Junhao Sun, Tianluo Guo, Peiran Hu, Xin Zhou, Xiaolong Jia, Xudong Yan, Zhihua Xu, Hongxing Shan, Huiqiang Zhao, Lu Miao, Shuai Ma, Bin Yuan, Hengji Li, Guilan Gou, Chao Zhu, Dazhi Yang, Junhua Tian, Yajun Deng, Jiancuo A

**Affiliations:** 1 Graduate School of Qinghai University, Xining, Qinghai, China; 2 Department of Spine Surgery, Qinghai Red Cross Hospital, Xining, Qinghai, China; 3 School of Medicine, Shenzhen University, Shenzhen, Guangdong, China; 4 Nanshan Hospital Affiliated to Shenzhen University, Shenzhen, Guangdong, China; 5 The Affiliated Jiangning Hospital of Nanjing Medical University, Nanjing, Jiangsu, China; 6 The 990th Hospital of the Chinese People’s Liberation Army Joint Logistics Support Force, Zhumadian, Henan, China; 7 Department of Orthopedics, Xi’an Daxing Hospital, Xi’an, Shaanxi, China

**Keywords:** bone–muscle metabolism, chronic low back pain, lumbar decompression surgery, machine learning, nutritional status

## Abstract

**Objective:**

This study aimed to investigate the associations of preoperative nutritional status, bone–muscle metabolic abnormalities, and paraspinal muscle degeneration with chronic low back pain (CLBP) after lumbar decompression surgery. A multicenter predictive model was developed to improve preoperative risk stratification, enable early identification of high-risk patients, and support individualized perioperative management.

**Methods:**

A total of 2,333 patients who underwent unilateral biportal endoscopic (UBE) decompression surgery at five centers were retrospectively enrolled. The cohort was divided into a training set, an internal validation set, and an external test set. Demographic characteristics, laboratory variables, and imaging parameters of the paraspinal muscles were collected. Multivariable logistic regression analysis was performed to identify independent factors associated with postoperative CLBP. Based on the selected variables, multiple machine learning models were developed. Model performance was evaluated using receiver operating characteristic (ROC) analysis, calibration curves, and decision curve analysis. In addition, model interpretability analyses were conducted to assess the contributions of key variables to the predictions.

**Results:**

Multivariable logistic regression analysis identified age, albumin (Alb), calcium (Ca), alkaline phosphatase (ALP), psoas muscle index (PMI), multifidus fat infiltration (MF FI), and erector spinae fat infiltration (ES FI) as independent factors associated with postoperative CLBP. Among the predictive models, machine learning models showed better discrimination than the traditional logistic regression model. This finding suggests that machine learning may be more effective in capturing complex nonlinear relationships associated with postoperative CLBP. The ExtraTrees model showed the best performance, with area under the curve (AUC) values of 0.834 in the internal validation set and 0.816 in the external test set. These values were higher than those of the logistic regression model. The model also showed good calibration, with Brier scores of 0.168 and 0.171 in the internal validation and external test sets, respectively, and provided stable clinical net benefit. Further analysis showed that age, PMI, and paraspinal muscle fat infiltration were the most important predictors of postoperative CLBP. A web-based calculator was subsequently developed to improve the clinical applicability of the model.

**Conclusion:**

Preoperative nutritional insufficiency, bone–muscle metabolic abnormalities, and paraspinal muscle degeneration were closely associated with CLBP after lumbar decompression surgery. A predictive model integrating these factors showed good discriminative ability for predicting postoperative pain risk. Preoperative assessment based on these variables may help identify high-risk patients at an early stage. It may also guide strategies for nutritional optimization, bone–muscle metabolism management, and perioperative rehabilitation, thereby supporting individualized perioperative care.

## 1 Introduction

Lumbar decompression surgery is widely used for the treatment of degenerative lumbar disorders. However, a substantial proportion of patients continue to experience chronic low back pain (CLBP) after surgery. This persistent pain has become a major factor limiting the overall benefit of surgery (1, 2). Although decompression can effectively relieve neural compression, postoperative pain cannot always be explained solely by structural abnormalities. More complex local and systemic factors are likely to contribute to this process. Therefore, identification of the key risk factors for postoperative CLBP is clinically important for optimizing perioperative management and improving patient outcomes. In recent years, paraspinal muscle degeneration has been recognized as an important contributor to postoperative pain. Fat infiltration and functional atrophy of the multifidus and erector spinae muscles may reduce dynamic spinal stability and alter load distribution. As a result, persistent pain may be promoted (3). In addition, growing evidence suggests that systemic factors should also be taken into account. Malnutrition may impair postoperative recovery through adverse effects on muscle mass, inflammatory regulation, and tissue repair capacity (4, 5). Abnormal bone–muscle metabolism may further aggravate spinal dysfunction by reducing muscle performance and weakening skeletal support (6). Collectively, these factors may contribute to postoperative pain through concurrent disturbances in muscle and bone metabolism. However, most existing studies have focused on single indicators, such as imaging changes in the paraspinal muscles. Integrated analyses of nutritional status, bone–muscle metabolism, and paraspinal muscle structural characteristics remain limited (7). More importantly, current risk assessment for CLBP after lumbar decompression surgery still relies primarily on clinical features or isolated imaging markers. An integrated evaluation of local structural alterations and systemic metabolic status remains lacking. This one-dimensional assessment strategy does not adequately capture the complex pathophysiological basis of postoperative pain. It also limits the accuracy of risk stratification and weakens its value for clinical decision-making. Therefore, development of a multidimensional predictive tool that integrates paraspinal muscle characteristics with systemic biological status is important for accurate risk assessment. Against this background, this multicenter study systematically evaluated the associations of nutritional status, bone–muscle metabolic abnormalities, and paraspinal muscle parameters with CLBP after lumbar decompression surgery. A machine learning–based predictive model was then developed to better capture complex nonlinear relationships. In addition, Shapley Additive Explanations (SHAP) and Local Interpretable Model-Agnostic Explanations (LIME) were used to interpret the model output at both the global and individual levels. Restricted cubic spline analysis(RCS)was further performed to characterize nonlinear associations between key variables and the outcome. Through this approach, predictive performance was improved, while clinical interpretability was maintained. This study was designed to provide a reliable basis for risk stratification and individualized intervention in patients with postoperative CLBP.

## 2 Materials and methods

### 2.1 Patient selection

**FIGURE 1:**
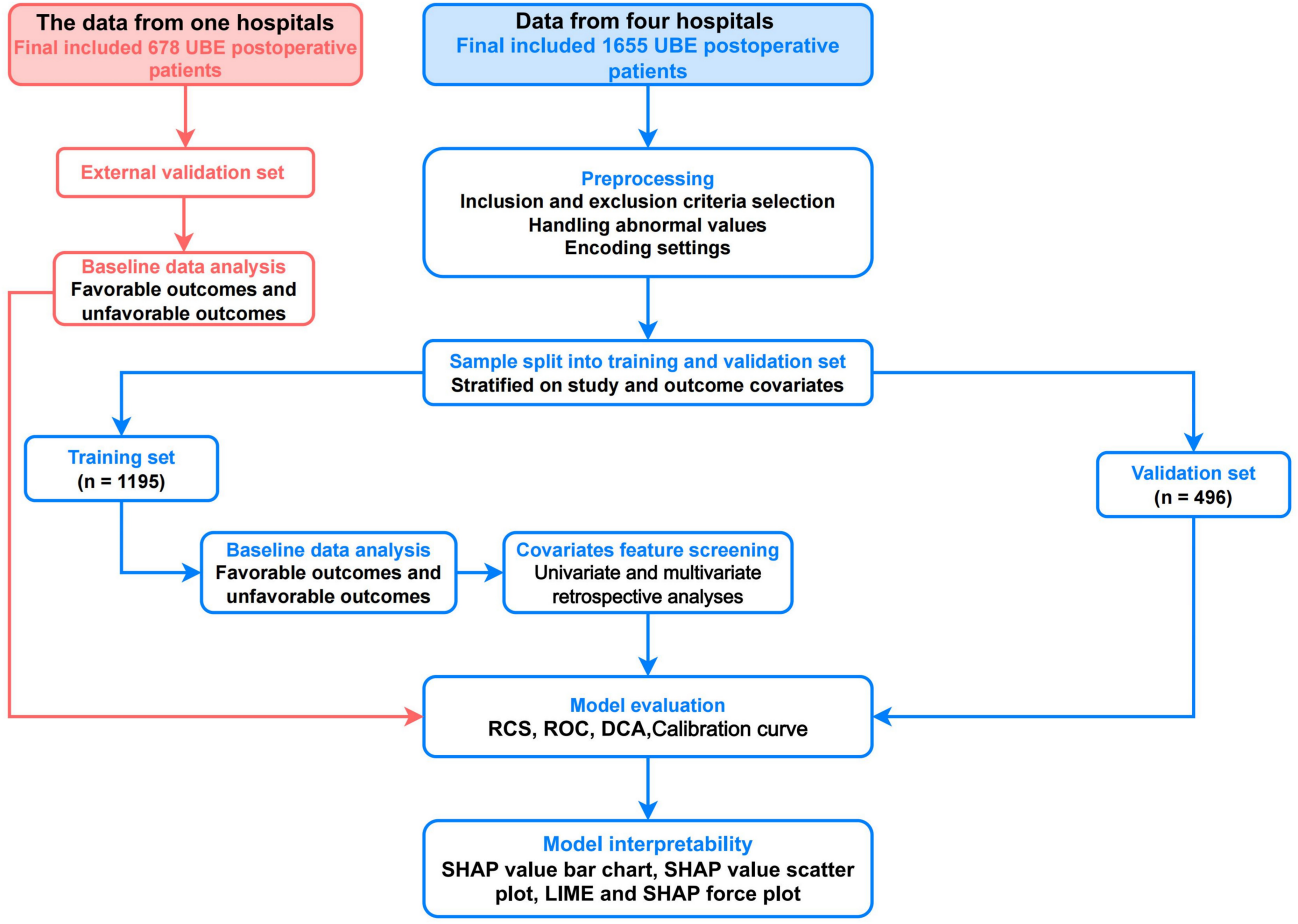
Flowchart depicts the patient’s enrollment process in the study.

A total of 2333 patients with lumbar disc herniation (LDH) who underwent unilateral biportal endoscopic (UBE) decompression surgery between January 2021 and December 2024 were retrospectively included in this study. The patients were recruited from five centers: Qinghai Red Cross Hospital, Xi’an Daxing Hospital, Jiangning People’s Hospital of Nanjing, No. 990 Hospital of the Joint Logistics Support Force of the Chinese People’s Liberation Army, and Shenzhen Nanshan District People’s Hospital. The inclusion criteria were as follows: (1) single-level lumbar disc herniation, with or without sensory disturbance in the distribution of the affected nerve root; (2) typical unilateral radicular pain, numbness, or muscle weakness in the lower extremity corresponding to the affected nerve root, with no significant improvement after 3 months of standard conservative treatment; (3) consistency between the clinical presentation and imaging findings; and (4) complete follow-up data. The exclusion criteria were as follows: (1) a history of revision surgery at the same level; (2) the presence of other spinal disorders that could substantially affect postoperative pain outcomes, such as ankylosing spondylitis, tumors, fractures, or tuberculosis; (3) incomplete imaging data; (4) instability at the target level requiring fusion and fixation; (5) loss to follow-up or incomplete outcome data; (6) long-term regular use of analgesics before surgery; (7) pain predominantly attributed to psychological factors; and (8) postoperative pain that could be explained by a definite organic cause, such as infection, dural tear, or recurrent disc herniation. The study flowchart is presented in Figure 1. All procedures were performed by five senior spine surgeons with extensive experience in minimally invasive techniques. A standardized surgical procedure was adopted to minimize the effect of inter-operator variation on outcomes. The study was coordinated by Qinghai Red Cross Hospital and approved by the ethics committees of all participating centers (approval numbers: KY-2025-154, LW2025-026, kY-2025-073001, 2025-03-090-K01, and KY-2025-76). The study was conducted in accordance with the Declaration of Helsinki. According to predefined criteria, the patients were classified into a CLBP group and a non-CLBP group. In addition, to reduce heterogeneity in this multicenter retrospective study, consistency measures were implemented during the design, data collection, and analysis phases. All participating centers used uniform inclusion and exclusion criteria, a standardized outcome definition, and a consistent surgical procedure.

### 2.2 Clinical characteristics

During the selection of potential predictive variables, an extensive literature review was conducted to identify factors previously shown to be important for outcome prediction. Based on the literature review and the predefined inclusion and exclusion criteria, a total of 35 variables were ultimately included in the study. Demographic variables included age, sex, body mass index (BMI), smoking history, alcohol consumption history, and operative duration. Imaging variables included the level and type of disc herniation (HT), as well as muscle-related parameters, including the psoas muscle index (PMI), functional cross-sectional area of the multifidus (MFfCSA), functional cross-sectional area of the erector spinae (ES fCSA), multifidus fat infiltration (MFFI), and erector spinae fat infiltration (ESFI). Laboratory variables included albumin (Alb), globulin, alkaline phosphatase (ALP), alanine aminotransferase (ALT), aspartate aminotransferase (AST), gamma-glutamyl transpeptidase (GGT), serum creatinine (Scr), uric acid (UA), C-reactive protein (CRP), lactate dehydrogenase, white blood cell count (WBC), lymphocyte count (LYMNO), monocyte count (MONO), neutrophil count (NENO), platelet count (PLT), red blood cell count (RBC), hemoglobin (Hb), and electrolyte and trace element indices, including calcium (Ca), potassium (K), sodium (Na), phosphorus (P), and iron (Fe). The outcome was defined as persistent low back pain lasting for more than 6 months after surgery, with a visual analog scale (VAS) score greater than 4. Patients with pain attributable to definite organic causes, such as infection, dural tear, or recurrent disc herniation, and those whose pain was predominantly attributed to psychological factors were excluded (8–13). In addition, to reduce potential bias related to differences in preoperative disease severity, the preoperative low back pain VAS score, lower extremity VAS score, and Oswestry Disability Index (ODI) were collected. These variables were used to assess the potential effect of baseline symptom severity on postoperative pain outcomes.

### 2.3 Extraction of radiologic features

**FIGURE 2:**
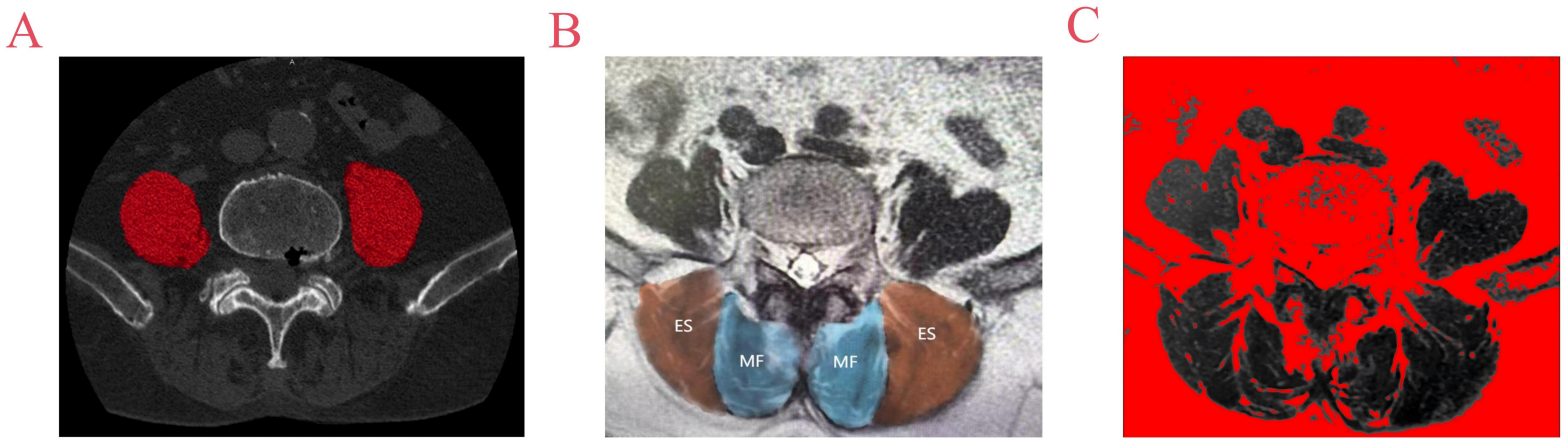
Imaging-based muscle measurements. **(A)** Manual segmentation of bilateral psoas muscles on axial CT at L4 (red) for CSA and PMI calculation. **(B)** ROI delineation of the paraspinal muscles on axial MRI at the inferior L4 endplate, including MF and ES. **(C)** Threshold-based segmentation in ImageJ to quantify FI; red mask indicates pixels meeting the preset threshold.

**FIGURE 3:**
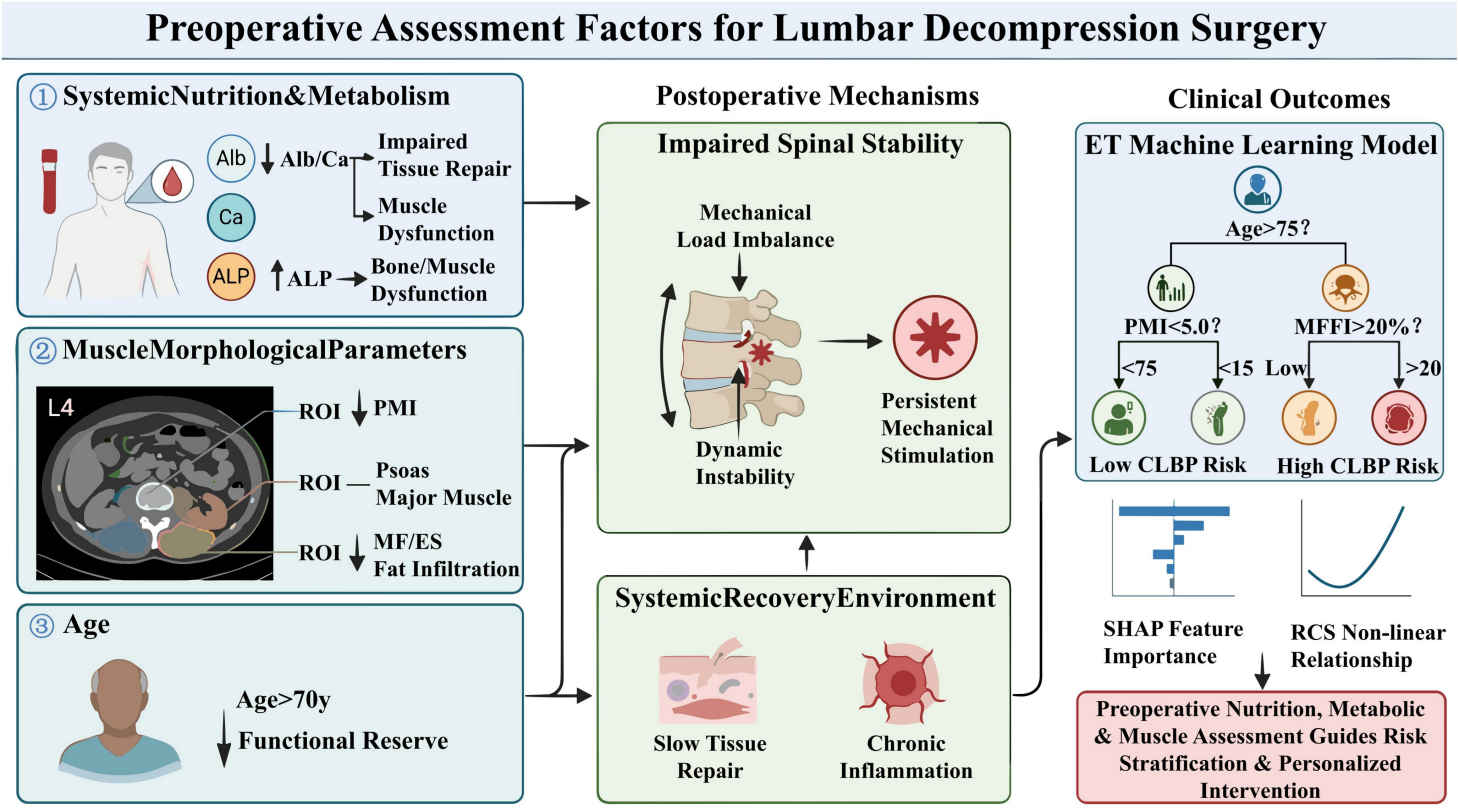
Conceptual framework of preoperative assessment and prediction of postoperative CLBP following lumbar decompression surgery.

Psoas major parameters were extracted from CT images. At the L4 level, the bilateral psoas major muscles were manually segmented using ImageJ with a standardized threshold of -29 to +150 HU. The CSA was then calculated (Figure 2A). The PMI was defined as the combined bilateral psoas CSA divided by height squared and was expressed as cm^2^/m^2^. The formula was as follows: PMI = bilateral psoas CSA/height^2^. Paraspinal muscle parameters were extracted from MRI images. At the level of the inferior endplate of L4, ROIs were manually delineated for the multifidus and erector spinae muscles. Each ROI was traced point by point along the outer border of the muscle. Surrounding adipose tissue was excluded as much as possible to ensure that the measured region accurately reflected muscle morphology and size (Figure 2B). After delineation, the corresponding CSA values were automatically generated, and FI was further assessed. All imaging measurements were performed twice, and the mean values were used for subsequent analysis. The FI area was measured in ImageJ using a threshold-based segmentation method, with the threshold set at 90–255 on the 8-bit grayscale scale. Pixel areas that met the threshold criteria were displayed as a red mask, and the fat areas of the multifidus and erector spinae muscles were calculated accordingly (Figure 2C). The FCSA was defined as the total CSA minus the fat area. The resulting value was then normalized by height squared and expressed as cm^2^/m^2^. The degree of FI was defined as the proportion of fat area to total muscle area and was expressed as a percentage. All imaging data were independently evaluated by three experienced senior spine surgeons who were blinded to the patients’ clinical characteristics and outcome data. In cases of disagreement, consensus was reached through discussion. The overall study workflow is shown in Figure 3.

### 2.4 Statistical analysis

Data were analyzed using SPSS version 27.0, and models were constructed using Python 3.10. The normality of continuous variables was assessed using the Shapiro–Wilk test. Normally distributed variables are presented as the mean ± standard deviation (SD), whereas non-normally distributed variables are presented as the median and interquartile range (IQR). Between-group comparisons were performed using analysis of variance (ANOVA) for normally distributed variables and the Kruskal–Wallis test for non-normally distributed variables. Categorical variables are reported as frequencies and percentages and were compared using Pearson’s chi-square test or Fisher’s exact test, as appropriate. Before model development, candidate predictors were selected based on previous literature, clinical relevance, and the inclusion and exclusion criteria of this study. A total of 35 candidate variables were included, comprising demographic characteristics, laboratory indices, and imaging-derived muscle parameters. In the training cohort, univariable logistic regression was first performed for all candidate variables. Variables with *P* < 0.05 in the univariable analysis were then entered into a multivariable logistic regression model. Finally, seven variables—age, Alb, Ca, ALP, PMI, MF FI, and ES FI—were retained for subsequent machine-learning model construction based on the multivariable results, statistical significance, clinical interpretability, and model stability. Multicollinearity among the candidate variables was assessed using the variance inflation factor (VIF). None of the seven variables included in the final model showed evidence of substantial multicollinearity; therefore, all were retained for model development. Regarding missing data, data completeness was strictly controlled during patient screening. Patients were required to have complete follow-up data, and cases with incomplete imaging data, loss to follow-up, or incomplete outcome data were excluded. Consequently, all patients included in the final model analysis had complete key clinical, laboratory, imaging, and outcome variables. Multiple imputation or other missing-value imputation methods were therefore not applied, and model development and validation were performed using the complete dataset. Model performance was comprehensively evaluated using the area under the receiver operating characteristic curve (AUC), accuracy, sensitivity, specificity, precision, and F1 score. The optimal model was validated in both an internal validation set and an independent external testing cohort from a different center to assess its generalizability and robustness across different data distributions. Calibration performance was evaluated using the Brier score and calibration curves, and the calibration intercept and calibration slope were calculated. Decision curve analysis (DCA) was further used to assess the net clinical benefit of the model across different threshold probabilities, thereby evaluating its potential clinical utility. To improve clinical interpretability, the optimal model was further analyzed using explainable artificial intelligence methods. At the global level, SHAP were used to quantify the overall contribution of each variable to model predictions and to identify key risk factors. At the local level, LIME were applied to explain predictions for individual patients and reveal patient-specific drivers of risk. In addition, RCS regression was used for modeling and visualization to further explore potential nonlinear associations between key continuous variables and the risk of postoperative CLBP.

## 3 Results

### 3.1 ICC consistency evaluation results

The repeatability and reliability of the imaging parameters were evaluated using the intraclass correlation coefficient (ICC) (Table 1). All imaging parameters included in the analysis demonstrated good interobserver agreement. The ICC values were 0.873 for PMI (95% CI, 0.849–0.894), 0.843 for MF FI (95% CI, 0.813–0.869), and 0.879 for ES FI (95% CI, 0.856–0.900). The ICC values for MF FCSA and ES FCSA were 0.841 (95% CI, 0.811–0.867) and 0.862 (95% CI, 0.836–0.885), respectively. All ICC analyses were statistically significant (all *P* < 0.001), indicating that the imaging measurement methods used in this study had high reproducibility and reliability.

**TABLE 1:** Reliability analysis of imaging-derived parameters.

Parameter	ICC Value	95%CI		t	*P*
		Lower	Upper		
PMI	0.873	0.849	0.894	21.655	<0.001
MF FI	0.843	0.813	0.869	17.189	<0.001
ES FI	0.879	0.856	0.900	22.865	<0.001
MF FCSA	0.841	0.811	0.867	16.817	<0.001
ES FCSA	0.862	0.836	0.885	19.796	<0.001

### 3.2 Patient characteristics

A total of 2,333 patients from five medical centers were included in this study. Among these patients, 1,655 from four centers (Qinghai Red Cross Hospital, Xi’an Daxing Hospital, No. 990 Hospital of the Joint Logistics Support Force of the Chinese People’s Liberation Army, and Shenzhen Nanshan District People’s Hospital) were randomly assigned to the training cohort (*n* = 1159) and the validation cohort (*n* = 496) at a 7:3 ratio. Patients from Jiangning People’s Hospital of Nanjing were included as an independent external test cohort (*n* = 678). The baseline characteristics of the patients are presented in Table 2. To minimize potential bias arising from differences in preoperative disease severity, preoperative low back pain VAS, leg pain VAS, and ODI were collected for all patients. No significant differences were observed among the cohorts (all *P* > 0.05).

**TABLE 2:** Patient demographics and baseline characteristics.

Variables	Train set (*n* = 1159)	Validation set (*n* = 496)	Test set (*n* = 678)	Statistic	*P*
Age, years	69.44 ± 10.62	70.02 ± 10.41	69.87 ± 9.84	*F* = 0.69	0.500
Gender, n(%)				χ^2^ = 1.17	0.557
Male	587 (50.65)	241 (48.59)	351 (51.77)		
Female	572 (49.35)	255 (51.41)	327 (48.23)		
BMI, kg/m^2^	25.96 ± 5.94	26.49 ± 5.99	26.56 ± 5.74	*F* = 2.79	0.062
Drink, n(%)	712 (61.43)	293 (59.07)	394 (58.11)	χ^2^ = 2.17	0.337
Smoke, n(%)	615 (53.06)	259 (52.22)	350 (51.62)	χ^2^ = 0.37	0.831
Herniation type, n(%)				χ^2^ = 1.68	0.431
Central	489 (42.19)	201 (40.52)	300 (44.25)		
Lateral	670 (57.81)	295 (59.48)	378 (55.75)		
Alb, g/dL	3.98 ± 0.31	3.97 ± 0.33	3.96 ± 0.32	*F* = 0.67	0.510
Ca, mg/dL	9.28 ± 0.37	9.25 ± 0.38	9.27 ± 0.36	*F* = 1.04	0.353
ALP, U/L	75.24 ± 23.30	78.29 ± 40.35	77.14 ± 25.82	*F* = 2.28	0.102
Globulin, g/dL	3.16 ± 0.53	3.21 ± 0.55	3.16 ± 0.48	*F* = 2.31	0.099
UA, mg/dL	5.57 ± 1.36	5.50 ± 1.44	5.64 ± 1.46	*F* = 1.50	0.223
Scr, mg/dL	0.92 ± 0.32	0.95 ± 0.34	0.93 ± 0.33	*F* = 0.89	0.411
CRP, mg/dL	0.66 ± 1.06	0.72 ± 1.80	0.74 ± 1.35	*F* = 0.98	0.374
LDH, U/L	141.60 ± 32.61	138.50 ± 34.11	140.64 ± 33.96	*F* = 1.51	0.222
ALT, U/L	27.07 ± 25.09	26.77 ± 20.81	26.76 ± 21.58	*F* = 0.05	0.950
AST, U/L	26.15 ± 18.44	27.19 ± 21.02	25.39 ± 14.25	*F* = 1.45	0.235
GGT, U/L	44.60 ± 83.25	35.64 ± 40.32	39.52 ± 69.94	*F* = 2.94	0.053
Hb, g/dL	14.09 ± 1.48	13.97 ± 1.52	14.12 ± 1.51	*F* = 1.45	0.235
PLT, × 10^9^/L	234.72 ± 49.33	232.58 ± 52.14	240.96 ± 49.95	*F* = 4.84	0.008
RBC, × 10^12^/L	4.67 ± 0.55	4.67 ± 0.50	4.66 ± 0.54	*F* = 0.09	0.916
WBC, × 10^9^/L	7.55 ± 2.52	7.60 ± 2.24	7.67 ± 2.43	*F* = 0.48	0.618
MONO, × 10^9^/L	0.58 ± 0.20	0.57 ± 0.19	0.57 ± 0.20	*F* = 0.48	0.618
NENO, × 10^9^/L	4.56 ± 1.67	4.39 ± 1.70	4.52 ± 1.82	*F* = 1.59	0.205
LYMNO, × 10^9^/L	2.23 ± 1.09	2.25 ± 2.08	2.19 ± 0.84	*F* = 0.33	0.716
K, mmol/L	4.07 ± 0.38	4.09 ± 0.38	4.09 ± 0.40	*F* = 0.67	0.512
Na, mmol/L	138.65 ± 2.76	138.74 ± 2.70	138.68 ± 2.62	*F* = 0.18	0.834
P, mg/dL	3.74 ± 0.63	3.60 ± 0.59	3.71 ± 0.60	*F* = 8.33	< 0.001
Fe, μg/dL	78.74 ± 32.70	78.90 ± 31.15	78.29 ± 32.06	*F* = 0.06	0.940
PMI, cm^2^/m^2^	4.96 ± 1.49	4.98 ± 1.48	4.98 ± 1.50	*F* = 0.04	0.956
MF FI, %cm^2^/m^2^	21.07 ± 4.97	21.13 ± 4.85	21.05 ± 5.13	*F* = 0.04	0.962
ES FI, %cm^2^/m^2^	16.87 ± 4.01	16.81 ± 4.16	16.70 ± 4.04	*F* = 0.38	0.687
MF fCSA,%	3.30 (2.91,3.71)	3.23 (2.89,3.68)	3.29 (2.89,3.72)	χ^2^ = 1.68	0.432
ES fCSA,%	10.51 (9.16,12.25)	10.50 (9.14,12.01)	10.35 (9.06,12.12)	χ^2^ = 1.86	0.394
Operation time, min	104.59 ± 4.93	104.28 ± 4.91	94.99 ± 11.27	*F* = 402.45	< 0.001
Level of herniated disc, n(%)				χ^2^ = 3.71	0.446
L3/4	123 (10.61)	52 (10.48)	77 (11.36)		
L4/5	736 (63.50)	311 (62.70)	401 (59.14)		
L5/S1	300 (25.88)	133 (26.81)	200 (29.50)		
Preop ODI,%	65.86 ± 6.38	66.14 ± 6.87	65.40 ± 5.47	*F* = 2.16	0.115
Preop Leg VAS	7.36 ± 0.76	7.40 ± 0.73	7.31 ± 0.78	*F* = 1.97	0.140
Preop Lumbar VAS	6.67 ± 0.80	6.65 ± 0.81	6.74 ± 0.85	*F* = 2.70	0.067

### 3.3 Feature selection

Based on univariate and multivariable analyses in the training cohort, age, Alb, Ca, ALP, PMI, MF FI, and ES FI were identified as independent predictors. These variables were subsequently included as the final features for ML model construction (Table 3).

**TABLE 3:** Univariate and multivariate analyses.

Variables	Univariate correlation analysis					Multivariate correlation analysis				
	β	S.E	*Z*	OR (95%CI)	*P*	β	S.E	*Z*	OR (95%CI)	*P*
Age	0.07	0.01	8.33	1.07 (1.05∼1.09)	< 0.001	0.07	0.01	8.05	1.07 (1.05∼1.09)	< 0.001
Gender										
Male				1.00						
Female	0.19	0.13	1.50	1.21 (0.94∼1.55	0.134					
Drink	–0.03	0.13	–0.22	0.97 (0.75∼1.25)	0.827					
Smoke	–0.03	0.13	–0.23	0.97 (0.76∼1.24)	0.820					
Herniation type	0.18	0.13	1.36	1.19 (0.93∼1.53)	0.173					
Alb	–1.12	0.22	-5.20	0.33 (0.21∼0.50)	< 0.001	–1.03	0.24	–4.31	0.36 (0.22∼0.57)	< 0.001
Ca	–0.77	0.18	–4.19	0.46 (0.32∼0.66)	< 0.001	–0.63	0.20	–3.16	0.53 (0.36∼0.79)	< 0.001
ALP	0.01	0.00	4.43	1.01 (1.01∼1.02)	< 0.001	0.01	0.00	3.47	1.01 (1.01∼1.02)	< 0.001
BMI	0.00	0.01	0.01	1.00 (0.98∼1.02)	0.993					
Globulin	0.05	0.12	0.44	1.05 (0.84∼1.33)	0.663					
UA	0.08	0.05	1.73	1.08 (0.99∼1.19)	0.084					
Scr	–0.20	0.20	–1.02	0.82 (0.55∼1.21)	0.307					
CRP	–0.02	0.06	–0.40	0.98 (0.86∼1.10)	0.688					
LDH	0.00	0.00	1.04	1.00 (1.00∼1.01)	0.298					
ALT	–0.00	0.00	–0.20	1.00 (0.99∼1.00)	0.845					
AST	0.00	0.00	1.33	1.00 (1.00∼1.01)	0.185					
GGT	0.00	0.00	0.23	1.00 (1.00∼1.00)	0.816					
WBC	0.03	0.02	1.18	1.03 (0.98∼1.08)	0.236					
LYMNO	0.01	0.06	0.26	1.01 (0.91∼1.13)	0.796					
MONO	0.11	0.32	0.33	0.90 (0.48∼1.68)	0.741					
PLT	0.00	0.00	1.51	1.00 (1.00∼1.00)	0.130					
RBC	0.13	0.11	1.14	1.14 (0.91∼1.43)	0.255					
Hb	0.03	0.04	0.80	1.03 (0.95∼1.13)	0.423					
K	0.16	0.17	0.99	1.18 (0.85∼1.63)	0.321					
P	–0.05	0.10	–0.49	0.95 (0.78∼1.16)	0.627					
Na	0.01	0.02	0.31	1.01 (0.96∼1.05)	0.756					
Fe	–0.00	0.00	–0.21	1.00 (1.00∼1.00)	0.832					
PMI	–0.36	0.05	–7.57	0.70 (0.64∼0.77)	< 0.001	–0.35	0.05	–6.64	0.71 (0.64∼0.78)	< 0.001
Operation time	0.00	0.01	0.19	1.00 (0.98∼1.03)	0.848
Level of herniated disc										
L3/4				1.00					
L4/5	0.33	0.22	1.53	1.39 (0.91∼2.14)	0.127				
L5/S1	0.11	0.24	0.45	1.11 (0.70∼1.78)	0.653				
MF FI	0.09	0.01	6.70	1.09 (1.06∼1.12)	< 0.001	0.09	0.01	6.33	1.10 (1.07∼1.13)	< 0.001
ES FI	0.13	0.02	7.90	1.14 (1.10∼1.18)	< 0.001	0.11	0.02	6.06	1.12 (1.08∼1.16)	< 0.001
MF fCSA	–0.14	0.13	–1.07	0.87 (0.67∼1.13)	0.285					
ES fCSA	0.03	0.03	0.75	1.03 (0.96∼1.10)	0.454					

### 3.4 Model performance and comparison

**FIGURE 4:**
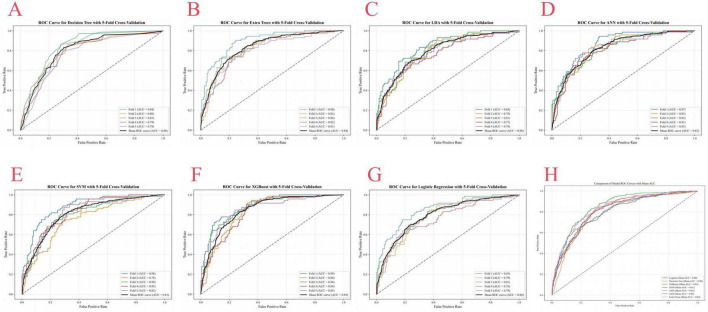
Comparison of ROC curves of different machine learning models under five-fold cross-validation. **(A)** Decision tree. **(B)** Extra trees. **(C)** Linear discriminant analysis. **(D)** Artificial neural network. **(E)** Support vector machine. **(F)** XGBoost. **(G)** Logistic regression. **(H)** Comparison of different machine learning models.

**FIGURE 5:**
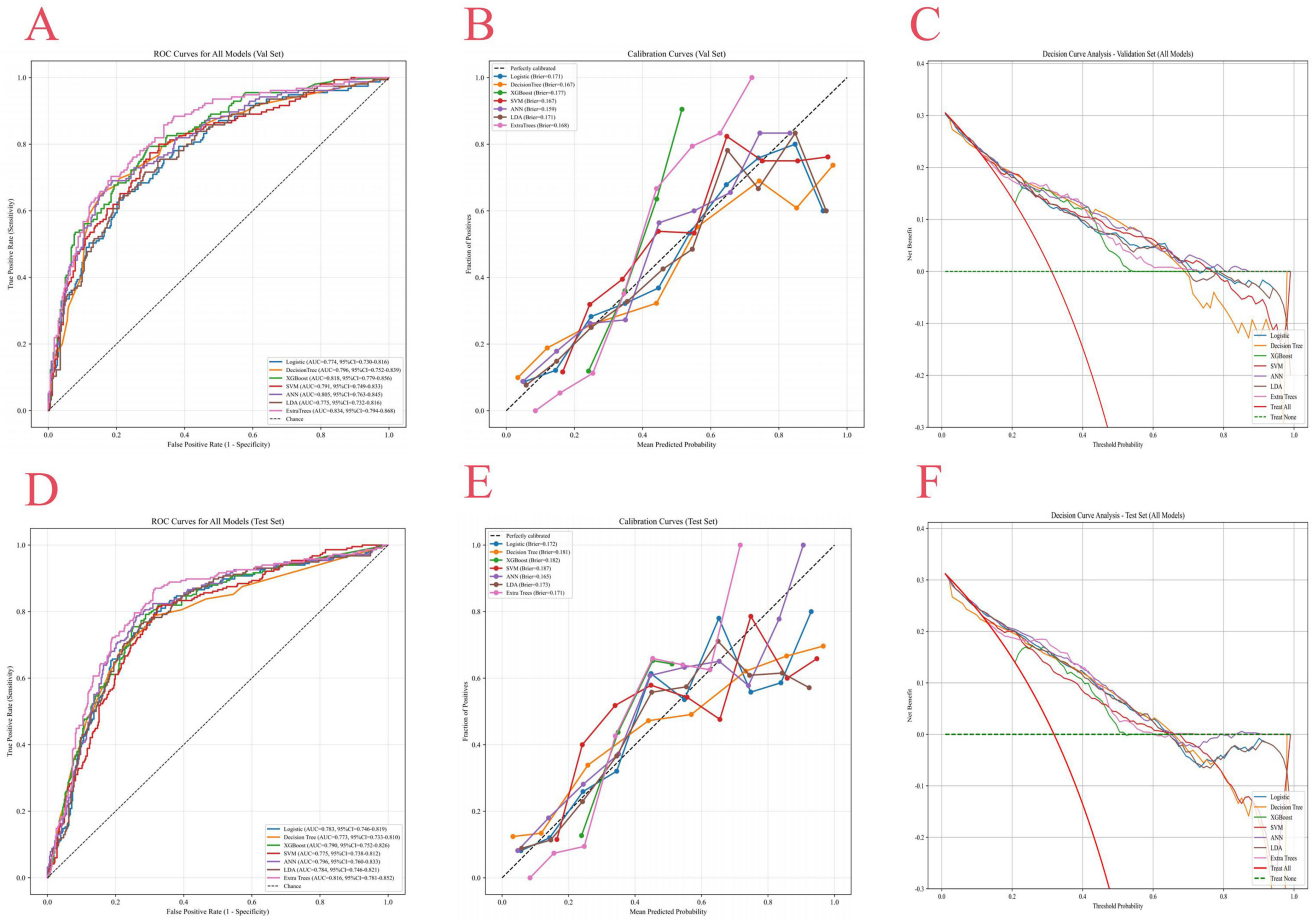
**(A)** ROC curves in the validation set. **(B)** Calibration curves in the validation set. **(C)** Decision curve analysis (DCA) in the validation set. **(D)** ROC curves in the external test set. **(E)** Calibration curves in the external test set. **(F)** Decision curve analysis (DCA) in the external test set.

During model development, repeated cross-validation was applied to seven candidate models to improve generalizability and reduce the risk of overfitting. Overall, the ExtraTrees (ET) model showed the most stable performance and the best overall predictive ability (Figure 4). After dataset partitioning, seven predictive models were developed and evaluated. Their performance in the internal validation and external test cohorts is summarized in Supplementary Table 1. In the internal validation cohort, the logistic regression (LR) model achieved an AUC of 0.774 (95% CI, 0.730–0.819). Most machine learning models showed better discrimination than the LR model. Among these models, the ET model performed best, with an AUC of 0.834 (95% CI, 0.796–0.872). The DeLong test showed that the ET model had significantly better discrimination than the LR model (*P* < 0.001). This finding suggests that machine learning methods may better capture complex nonlinear relationships. In the external test cohort, differences in AUC among the models were smaller than those in the internal validation cohort. Nevertheless, the ET model maintained good discrimination, with an AUC of 0.816, and its performance was not inferior to that of the LR model. This result indicates good generalizability of the ET model. Additional DeLong test results are provided in Supplementary Table 2. These results compare the statistical significance of AUC differences between each machine learning model and the LR model. Regarding classification performance, the ET model achieved a sensitivity of 0.703 and an F1 score of 0.649 in the validation cohort. These results indicate good identification of positive cases and balanced overall performance. In the external test cohort, the sensitivity and F1 score were 0.722 and 0.672, respectively, indicating similarly balanced performance. Calibration curve analysis showed good calibration of the ET model in both the validation and test cohorts. The Brier scores were 0.168 and 0.171, respectively, indicating good agreement between predicted probabilities and observed outcomes. Decision curve analysis (DCA) showed that the ET model provided a stable net benefit across threshold probabilities of approximately 0.05–0.75 in the validation cohort and 0.05–0.80 in the test cohort (Figure 5). These findings suggest that the ET model had stable probabilistic prediction performance and potential clinical utility across independent datasets.

### 3.5 Model interpretation

**FIGURE 6:**
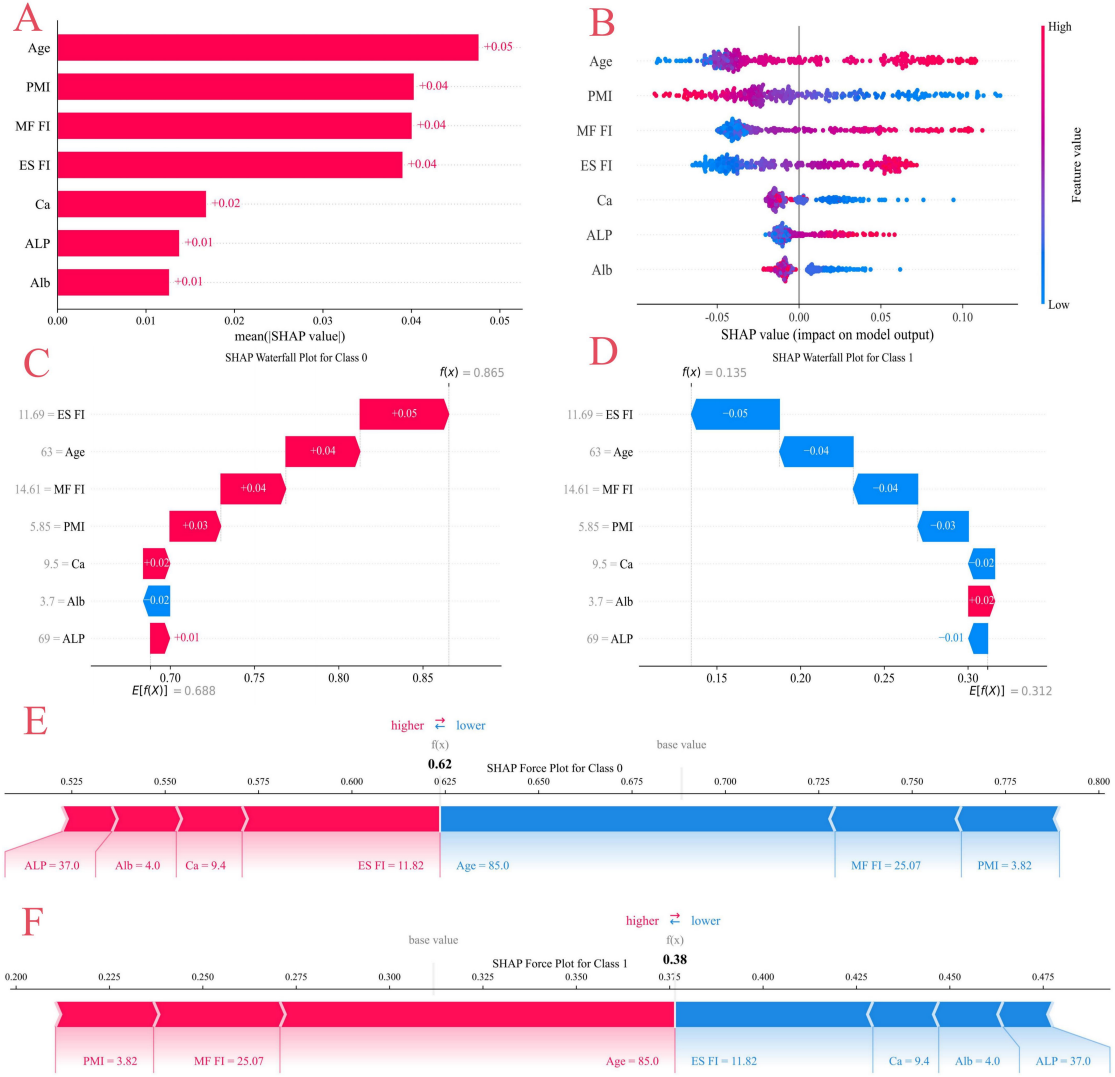
SHAP-based interpretation of the ET stacking model. **(A)** Global feature importance ranked by mean absolute SHAP values. **(B)** SHAP beeswarm plot showing the distribution of feature effects; color indicates feature value (low to high). **(C)** SHAP waterfall plot for a representative sample predicted as class 0 (no postoperative CLBP). **(D)** SHAP waterfall plot for the same sample predicted as class 1 (postoperative CLBP). **(E)** SHAP force plot for a representative sample predicted as class 1 (postoperative CLBP). **(F)** SHAP force plot for the same sample predicted as class 0 (no postoperative CLBP).

To further interpret the predictive mechanism of the ET model, SHAP analysis was performed to quantify the contribution of each feature to the model output (Figure 6). Feature importance analysis (Figure 6A) showed that age had the greatest influence on model predictions, followed by PMI, MF FI, and ES FI, whereas Ca, ALP, and Alb made relatively smaller contributions. The SHAP summary plot (Figure 6B) further illustrated the direction and distribution of the effects of each feature. Higher values of age, MF FI, and ES FI were associated with an increased risk. In contrast, lower PMI values were associated with a higher risk, indicating an inverse association with the outcome. Lower levels of Alb and Ca were associated with increased risk, whereas higher levels appeared to exert a protective effect. At the individual level, SHAP waterfall plots (Figures 6C,D) demonstrated that ES FI, age, and MF FI were the major contributors to increased risk in high-risk individuals. In contrast, in low-risk individuals, these factors contributed to a lower predicted risk. SHAP force plots (Figures 6E,F) further visualized the cumulative contribution of each feature in individual samples, showing how positive and negative effects jointly determined the final prediction. In addition, LIME was used for local interpretability analysis in a representative case (Supplementary Figure 1). In this case, the predicted probability of a favorable outcome was 0.87, whereas that of an unfavorable outcome was 0.13. Feature weights indicated that age ≤ 65 years contributed most strongly to a favorable prediction, whereas Alb ≤ 3.80 had a mildly negative effect.

### 3.6 Nonlinear associations between continuous variables and risk of adverse outcomes

**FIGURE 7:**
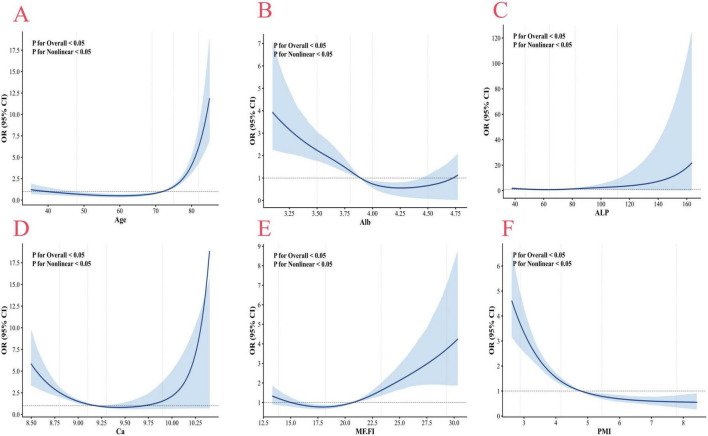
RCS analyses of continuous variables and postoperative CLBP risk: **(A)** Age. **(B)** Alb. **(C)** ALP. **(D)** Ca. **(E)** MF.FI. **(F)** PMI.

RCS analysis revealed significant nonlinear associations of age, Alb, ALP, Ca, MF FI, and PMI with outcome risk. For each variable, both the overall association and the nonlinear component were statistically significant (P for overall < 0.05; P for nonlinearity < 0.05). All RCS models were fitted using three knots, and the median value of each variable was used as the reference. The knot locations and reference values are presented in Figure 7. Specifically, age was nonlinearly and positively associated with outcome risk. Risk remained relatively stable in younger and middle-aged patients, increased gradually after approximately 70 years of age, and rose more markedly after 80 years of age. Alb was nonlinearly and inversely associated with outcome risk. Lower Alb levels were associated with higher risk, which decreased as Alb increased and then plateaued at approximately 4.0 g/dL. ALP was nonlinearly and positively associated with outcome risk. Risk changed only modestly at low to moderate ALP levels but increased progressively at higher levels, with a more pronounced increase at high ALP values. Ca showed an approximately U-shaped association with outcome risk, and the lowest risk was observed at approximately 9.2–9.5 mg/dL. Both lower and higher Ca levels were associated with an increased risk. MF FI was nonlinearly and positively associated with outcome risk. Risk was relatively low at low to moderate MF FI levels and increased progressively with higher MF FI. PMI was nonlinearly and inversely associated with outcome risk. Lower PMI levels were associated with higher risk, whereas risk decreased substantially as PMI increased and then plateaued at higher levels.

### 3.7 Development of an online calculator

**FIGURE 8:**
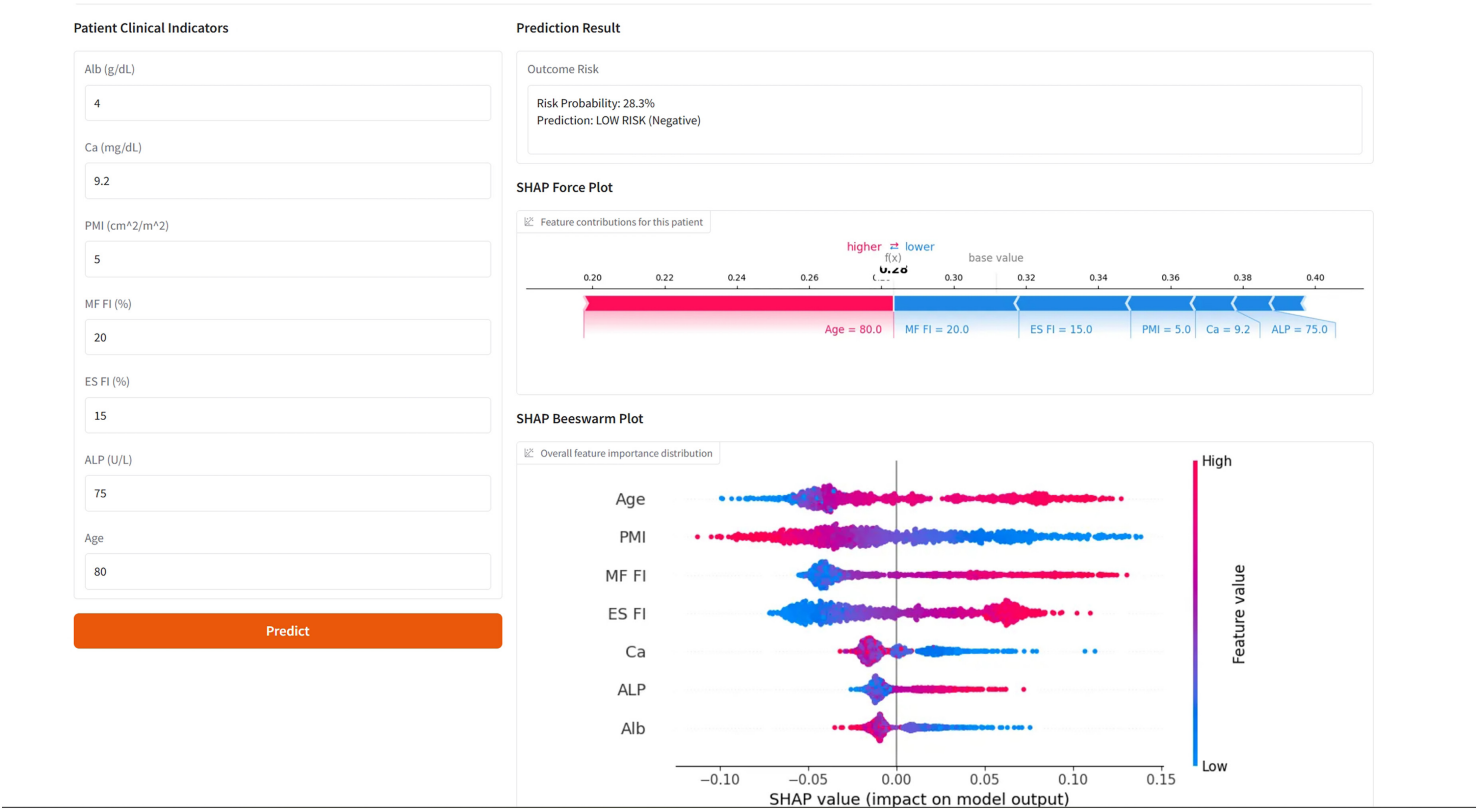
Interface of the online prediction calculator based on the ET model and visualization of SHAP-based interpretability results.

An online calculator based on the predictive model was developed.^1^ This tool enables clinicians to input patients’ clinical and imaging features to estimate the risk of postoperative chronic low back pain (CLBP) in individuals with lumbar disc herniation (LDH) (Figure 8).

## 4 Discussion

In recent years, clinical risk prediction tools have been widely used for prognostic assessment, perioperative risk stratification, and individualized clinical decision-making. These tools have played an important role in improving the identification of high-risk patients and optimizing treatment strategies. Most previous prediction models were developed using traditional statistical methods. In general, multiple potential predictors were incorporated into logistic regression models, and variables were weighted according to their regression coefficients to construct risk prediction systems. Although these approaches provide good interpretability and clinical readability, they usually rely on prespecified linear additive relationships. As a result, their ability to handle complex nonlinear associations, higher-order interactions, and highly heterogeneous clinical data may be limited (14). For complex outcomes such as chronic low back pain (CLBP) after lumbar decompression surgery, which is driven by multiple factors, traditional regression models may not fully capture the underlying risk structure. This limitation may become more apparent when a larger number of imaging, biochemical, and clinical variables are included. If the modeling strategy is not appropriately designed, the risk of overfitting may increase, and model performance may be overestimated. By contrast, machine learning methods can more flexibly identify complex data patterns and potential nonlinear relationships. Accordingly, increasing attention has been given to these methods in recent years for the prediction of postoperative complications and prognosis (15). The findings of the present study support this view. Compared with traditional logistic regression, the machine learning models showed better predictive performance in both the internal validation and external test cohorts. This finding suggests that machine learning may have greater potential for postoperative CLBP risk stratification. In addition, to minimize the influence of potential confounding factors and improve model generalizability, a large-sample, multicenter design was adopted in this study. This design improved sample representativeness and external validity. It also enhanced the reliability and reproducibility of the findings (16). More importantly, this study was not limited to improving model performance alone. Instead, the predictive framework was built on variables with clear clinical and biological relevance. The key indicators included in the model, such as age, paraspinal muscle parameters, and metabolism-related markers including Ca, characterized the risk basis of postoperative CLBP from multiple dimensions, including degenerative changes, local muscle structure, and systemic metabolic status. As a result, the model not only showed predictive value but also retained a clear pathophysiological basis for interpretation.

In this study, age was identified as the most important predictor and was closely associated with an increased risk of postoperative CLBP. This finding may reflect the cumulative effects of age-related degenerative changes. With advancing age, intervertebral disc degeneration, facet joint osteophyte formation, and paraspinal muscle fat infiltration tend to progress. These changes are often accompanied by sarcopenia, which may lead to impaired spinal biomechanics. As a result, local neural structures may remain susceptible to persistent mechanical stress or irritation after surgery, thereby contributing to pain persistence (17). In addition, a clear nonlinear relationship was observed between age and risk. Risk increased more rapidly after the age of 70, suggesting that compensatory and reparative capacities become markedly limited once physiological reserve declines beyond a certain threshold. Age-related functional decline often overlaps with muscle degeneration, further increasing the likelihood of persistent postoperative pain. These findings indicate that muscle status may play a key role in linking degenerative changes to postoperative pain (18). Previous studies have suggested that increased psoas muscle mass may be associated with a higher risk of recurrent lumbar disc herniation in middle-aged and older patients. As a spinal flexor, the psoas muscle may increase the mechanical load on the intervertebral disc during flexion, thereby accelerating degeneration (19). In addition, psoas muscle status has been shown to be closely associated with lumbar lordosis (20). Based on these findings, the present study further quantified this mechanism from the perspective of muscle reserve. PMI, which standardizes muscle CSA by body size, provides a more stable measure of core muscle reserve. The results showed that reduced PMI was significantly associated with an increased risk of postoperative CLBP, indicating that insufficient muscle reserve plays an important role in pain persistence. Unlike simple anatomical decompression, postoperative pain relief depends largely on restoration of dynamic spinal stability, which in turn relies on muscle integrity and functional reserve. From a biomechanical perspective, a lower PMI reflects a reduction in the active structures that contribute to spinal stability and load sharing. Consequently, greater reliance is placed on passive structures, increasing the likelihood of local stress concentration and persistent mechanical irritation (21). Under such conditions, even when neural compression has been adequately relieved, local tissues may remain under ongoing irritation because of insufficient dynamic support, thereby hindering complete pain resolution (22). Moreover, postoperative recovery is not solely a structural process but also involves re-establishment of neuromuscular control. A reduced PMI may reflect inadequate overall muscle reserve, making it more difficult for patients to develop effective trunk stabilization strategies during rehabilitation. This may adversely affect motor control and load distribution, thereby increasing the likelihood of persistent pain (23). Therefore, PMI reflects not only muscle quantity but also, to some extent, the potential for functional recovery. It should also be noted that muscle degeneration is not limited to a reduction in muscle mass; changes in muscle quality are equally important. The MF and ES play distinct yet complementary roles in spinal stability. The MF primarily contributes to segmental control, whereas the ES is more involved in postural maintenance and trunk extension (24). Increased FI in these muscles may therefore indicate not only structural degeneration but also impaired function at different levels of spinal control. When both segmental control and global postural stability are compromised, the spine’s ability to regulate load during daily activities is reduced. This may result in abnormal stress distribution and persistent tissue irritation (25). Therefore, persistent postoperative pain may not be solely attributable to residual neural irritation but may be more closely related to inadequate recovery of dynamic spinal stability. Even when adequate decompression has been achieved, repeated low-grade mechanical irritation may persist if paraspinal muscle function is not restored, thereby limiting pain relief (26). In addition, a nonlinear relationship was observed between FI and postoperative CLBP risk. When FI was mild, the muscle system appeared to retain a certain compensatory capacity. However, once a threshold was exceeded, the effect of muscle dysfunction on spinal stability became more pronounced. This finding suggests that a single muscle parameter may not fully capture the risk structure of postoperative CLBP and that comprehensive assessment of muscle quality may be more clinically meaningful. Overall, postoperative CLBP depends not only on adequate neural decompression but also on recovery of dynamic spinal stability. Beyond local muscle degeneration, this study also found that nutritional and metabolic indicators were closely associated with postoperative CLBP risk. This suggests that postoperative pain is influenced not only by local structural factors but also by the systemic recovery environment. Compared with imaging parameters, laboratory markers can provide additional information on tissue repair capacity, bone metabolic status, and neuromuscular function, thereby offering important insight into the underlying risk of postoperative CLBP. Alb, as a classical marker of nutritional status, is closely related to protein synthesis, inflammatory response, and tissue repair capacity. In this study, lower Alb levels were associated with an increased risk of postoperative CLBP, suggesting that inadequate nutritional reserve may impair postoperative tissue repair and functional recovery, thereby hindering pain relief (27). Recovery after lumbar decompression surgery depends not only on resolution of neural compression but also on soft tissue healing, inflammation control, and neuromuscular reconstruction, all of which may be influenced by nutritional status. It should be noted that Alb is not solely a marker of nutritional status. As a negative acute-phase protein, serum Alb may also be affected by systemic inflammation, infection, and perioperative stress-related inflammatory responses. Therefore, the observed association between lower Alb and an increased risk of postoperative CLBP may reflect inadequate nutritional reserve. It may also be partly confounded by underlying inflammatory status. In the present study, CRP was included as an inflammation-related variable. Patients with postoperative pain attributable to definite organic causes, including infection, dural tear, or recurrent disc herniation, were excluded during screening. Nevertheless, residual confounding from subclinical or unrecognized inflammatory conditions could not be fully excluded. Furthermore, the associations of Ca and ALP with postoperative CLBP suggest a potential role for bone metabolism in postoperative outcomes. Ca is involved in bone homeostasis, neuromuscular excitability, and muscle contraction, whereas ALP reflects bone turnover activity to some extent (28). Abnormalities in these markers may indicate imbalances in the bone–muscle metabolic environment, which may impair spinal load adaptation and recovery. Importantly, these systemic factors are unlikely to act independently of local muscle changes. Instead, they may interact with reduced muscle reserve and increased FI, thereby amplifying the risk of persistent postoperative pain. These findings suggest that the risk of postoperative CLBP is not solely determined by the adequacy of decompression but rather reflects the combined effects of local structural degeneration, reduced muscle reserve, and systemic nutritional and metabolic status. This observation has important clinical implications. Traditional preoperative assessments focus primarily on identification of the responsible segment and the degree of neural compression, whereas less attention is given to systemic recovery capacity, muscle reserve, and metabolic status. The key features identified in this study, including Alb, Ca, ALP, PMI, and paraspinal muscle FI, can all be obtained from routine preoperative examinations or imaging studies. Therefore, a risk assessment tool based on these variables may serve as a valuable supplement to standard preoperative evaluation. Moreover, the predictive framework developed in this study not only identifies risk but also suggests potential intervention strategies. For patients with low Alb or inadequate nutritional reserve, enhanced nutritional assessment and support may be considered. For those with abnormal Ca or ALP levels, further evaluation and optimization of bone metabolism may be warranted. For patients with low PMI or marked paraspinal muscle FI, greater emphasis may be placed on prehabilitation, core muscle training, and postoperative rehabilitation. In elderly patients, enhanced preoperative counseling and perioperative management may also be beneficial. Although this study does not directly demonstrate that these interventions improve outcomes, the findings suggest that prevention of postoperative CLBP should not rely solely on surgical decompression. Instead, it should be extended to comprehensive management of the patient’s overall recovery capacity. This perspective brings the predictive model closer to a clinical decision-support tool rather than a purely statistical model.

Several limitations should be acknowledged. First, the analysis was retrospective and was based on multicenter electronic medical record data. Although the sample size was relatively large, and training, internal validation, and independent external test cohorts were established to assess model stability, selection and information biases could not be fully excluded. Therefore, the findings should be interpreted primarily as evidence of risk associations rather than causal relationships. Second, although the outcome definition was clinically practical, it partly relied on pain scores and follow-up assessments. Differences among centers in follow-up procedures and in the interpretation of subjective scales may have introduced outcome misclassification. Third, metabolic indicators were defined using the first laboratory measurements obtained at admission. Moreover, all patients received standardized preoperative medical management, and surgery was performed only after blood glucose levels had been controlled within a relatively safe range. Thus, these indicators mainly reflected baseline metabolic status. They were insufficient to capture the potential effects of dynamic perioperative metabolic factors on postoperative pain outcomes, such as real-time glucose levels, glycemic variability, and hypoglycemic events. Future studies should incorporate continuous perioperative monitoring data and information on glucose-lowering regimens to enable a more refined assessment of modifiable metabolic risk factors. Fourth, although serum calcium and ALP were included as markers of bone metabolism, BMD, a direct indicator of bone quality, was not available. Low BMD often coexists with age-related muscle degeneration, paraspinal muscle fat infiltration, and degenerative spinal changes. Therefore, BMD may have confounded the associations among age, paraspinal muscle parameters, and postoperative chronic low back pain. Because BMD data were unavailable, the potential influence of bone mass status on the identified risk factors and model predictions could not be further evaluated. Future studies should include BMD measurements and comprehensive bone metabolism assessments to clarify the role of bone–muscle interactions in postoperative chronic low back pain. Fifth, the imaging and muscle-related parameters were mainly derived from static images and manual measurements. Although these parameters can reflect structural degeneration and muscle fat infiltration, they cannot fully capture dynamic biomechanical changes under different postural or loading conditions. Moreover, manual segmentation and measurement are limited by low efficiency, interobserver variability, and reduced cross-center reproducibility. Future studies may benefit from the integration of dynamic imaging, functional assessment, and automated segmentation techniques to improve efficiency and consistency. Sixth, regarding model applicability, the present study focused primarily on a specific surgical approach. Although the external test results suggested some degree of cross-center stability, the generalizability of the model to other surgical techniques remains uncertain. Prospective multicenter studies across different regions are needed to further evaluate model robustness and broader clinical applicability.

## 5 Conclusion

Preoperative nutritional insufficiency, bone–muscle metabolic abnormalities, and paraspinal muscle degeneration were closely associated with CLBP after lumbar decompression surgery. A predictive model integrating these factors showed good discriminative ability for predicting postoperative pain risk. Preoperative assessment based on these variables may help identify high-risk patients at an early stage. It may also guide strategies for nutritional optimization, bone–muscle metabolism management, and perioperative rehabilitation, thereby supporting individualized perioperative care.

## Data Availability

The data analyzed in this study is subject to the following licenses/restrictions: The datasets used and/or analyzed during the current study are available from the corresponding author on reasonable request. Requests to access these datasets should be directed to Jiancuo A, ajiancuo@126.com.

## Footnotes

1
https://huggingface.co/spaces/qq81273288/clinical-outcome-prediction
