# Supplementary material for: Impact of nutritional status and abnormal bone–muscle metabolism on chronic low back pain after lumbar decompression surgery: a multicenter predictive model study based on paraspinal muscle parameters
Source: Front Nutr. 2026 Jul 2;13:1848387. doi: 10.3389/fnut.2026.1848387 (PMC13372653; doi:10.3389/fnut.2026.1848387)
Supplement: Supplementary file 1 [file Table_1.docx]

| **Model** | **Dataset** | **AUC** | **Accuracy** | **Precision** | **Sensitivity** | **Specificity** | **F1** |
| --- | --- | --- | --- | --- | --- | --- | --- |
| Logistic | Test | 0.783 | 0.743 | 0.581 | 0.699 | 0.764 | 0.634 |
| Decision Tree | Test | 0.773 | 0.751 | 0.594 | 0.685 | 0.781 | 0.637 |
| XGBoost | Test | 0.79 | 0.742 | 0.587 | 0.639 | 0.79 | 0.612 |
| SVM | Test | 0.775 | 0.737 | 0.599 | 0.532 | 0.833 | 0.564 |
| ANN | Test | 0.796 | 0.767 | 0.617 | 0.708 | 0.794 | 0.659 |
| LDA | Test | 0.784 | 0.746 | 0.585 | 0.704 | 0.766 | 0.639 |
| Extra Trees | Test | 0.816 | 0.776 | 0.629 | 0.722 | 0.801 | 0.672 |
| Logistic | Val | 0.7743 | 0.7324 | 0.5604 | 0.6581 | 0.7661 | 0.6053 |
| Decision Tree | Val | 0.7957 | 0.7626 | 0.6034 | 0.6968 | 0.7924 | 0.6467 |
| XGBoost | Val | 0.8181 | 0.7586 | 0.5989 | 0.6839 | 0.7924 | 0.6386 |
| SVM | Val | 0.7912 | 0.7686 | 0.6587 | 0.5355 | 0.8743 | 0.5907 |
| ANN | Val | 0.8049 | 0.7706 | 0.6185 | 0.6903 | 0.807 | 0.6524 |
| LDA | Val | 0.7753 | 0.7243 | 0.5484 | 0.6581 | 0.7544 | 0.5982 |
| Extra Trees | Val | 0.8341 | 0.7626 | 0.6022 | 0.7032 | 0.7895 | 0.6488 |
