# Supplementary material for: Impact of nutritional status and abnormal bone–muscle metabolism on chronic low back pain after lumbar decompression surgery: a multicenter predictive model study based on paraspinal muscle parameters
Source: Front Nutr. 2026 Jul 2;13:1848387. doi: 10.3389/fnut.2026.1848387 (PMC13372653; doi:10.3389/fnut.2026.1848387)
Supplement: Supplementary file 2 [file Table_2.docx]

**Supplementary Table2. DeLong test comparisons between machine learning models and LR**

| **Comparison** | **Validation P value** | **Test P value** |
| --- | --- | --- |
| Decision Tree vs LR | 0.3218 | 0.2871 |
| XGBoost vs LR | 0.0163 | 0.0112 |
| SVM vs LR | 0.4422 | 0.5011 |
| ANN vs LR | 0.0231 | 0.0198 |
| LDA vs LR | 0.7906 | 0.8441 |
| Extra Trees vs LR | <0.001 | <0.001 |
