# Supplementary figures and images for: Impact of nutritional status and abnormal bone–muscle metabolism on chronic low back pain after lumbar decompression surgery: a multicenter predictive model study based on paraspinal muscle parameters
Source: Front Nutr. 2026 Jul 2;13:1848387. doi: 10.3389/fnut.2026.1848387 (PMC13372653; doi:10.3389/fnut.2026.1848387)

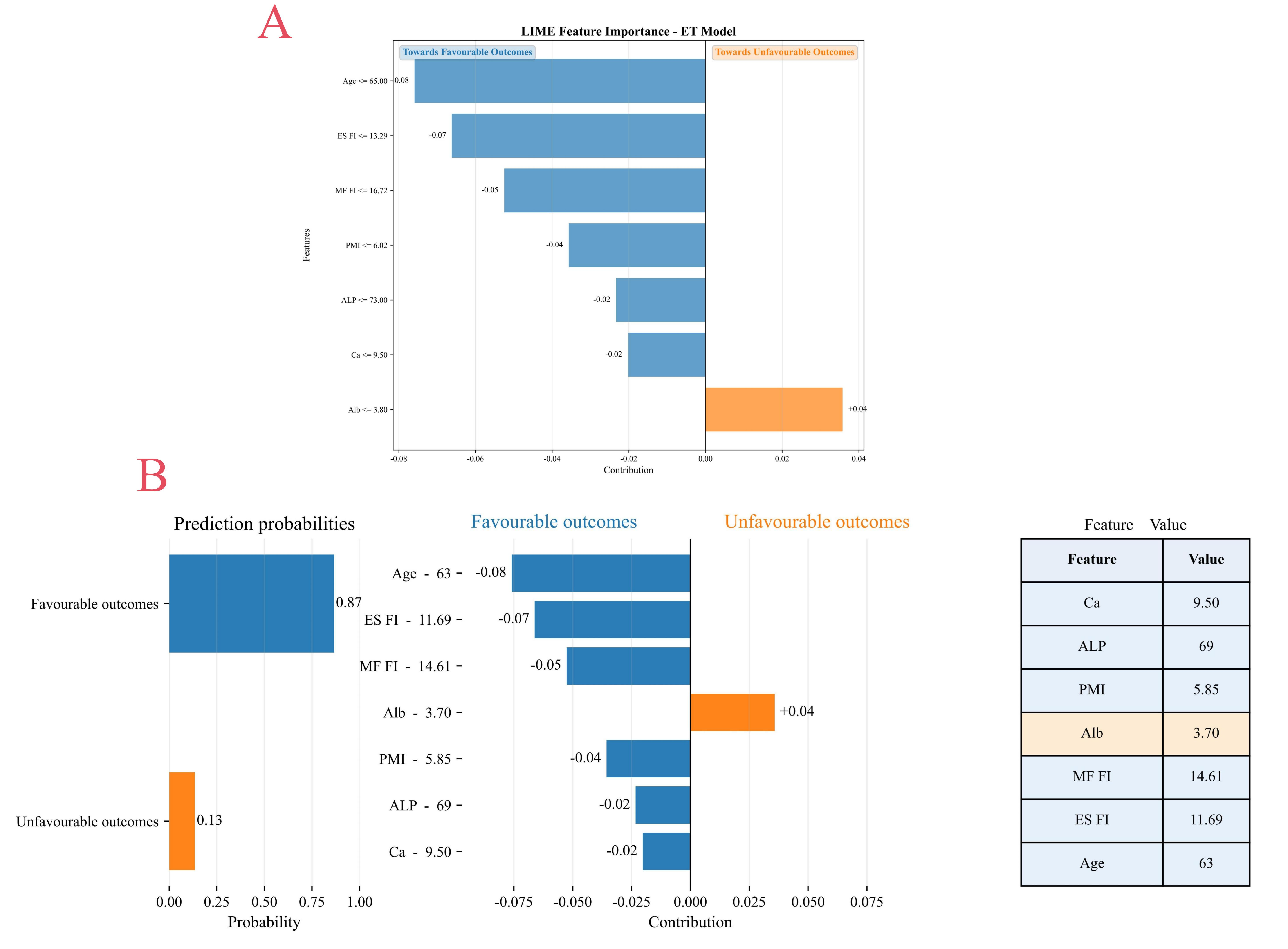

Supplement: Supplementary file 3 [file Image_1.jpeg]
